# Cost‐Performance Analysis of Perovskite Solar Modules

**DOI:** 10.1002/advs.201600269

**Published:** 2016-09-15

**Authors:** Molang Cai, Yongzhen Wu, Han Chen, Xudong Yang, Yinghuai Qiang, Liyuan Han

**Affiliations:** ^1^ Center for Green Research on Energy and Environmental MaterialsNational Institute for Materials ScienceTsukubaIbaraki305‐0047Japan; ^2^ State Key Laboratory of Metal Matrix CompositesShanghai Jiao Tong University800 Dong Chuan RD. Minhang DistrictShanghai200240PR China; ^3^ School of Mechatronic EngineeringChina University of Mining and TechnologyXuzhou221008JiangsuPR China

**Keywords:** cost analysis, perovskite solar cells, perspective

## Abstract

Perovskite solar cells (PSCs) are promising candidates for the next generation of solar cells because they are easy to fabricate and have high power conversion efficiencies. However, there has been no detailed analysis of the cost of PSC modules. We selected two representative examples of PSCs and performed a cost analysis of their productions: one was a moderate‐efficiency module produced from cheap materials, and the other was a high‐efficiency module produced from expensive materials. The costs of both modules were found to be lower than those of other photovoltaic technologies. We used the calculated module costs to estimate the levelized cost of electricity (LCOE) of PSCs. The LCOE was calculated to be 3.5–4.9 US cents/kWh with an efficiency and lifetime of greater than 12% and 15 years respectively, below the cost of traditional energy sources.

## 1 Introduction

The photovoltaic (PV) market has increased dramatically during recent decades. In 2014, there were about 40 GW of PV modules installed globally, 92% of which were crystalline silicon solar cells.^1^ Although the price of silicon modules has decreased dramatically, the cost of electricity produced by PVs is still higher than that of electricity supplied by conventional fossil fuels.^2^, ^3^ Hence, expansion of the PV market has relied on government support to a great extent in the past. For example, as a result of the policy of feed‐in tariffs,^4^ PV installations in Europe increased greatly at the beginning of this century.^5^ However, subsequent political policy adjustments have led to a considerable decline of PV installations in Europe.^6^ To consistently promote the PV market, there is an urgent need to establish a cost‐effective PV industry that can survive without government support.

The effort to lower costs has resulted in the development of many new PV technologies based on cheap materials and low‐cost processes, such as thin‐film silicon solar cells^7^ and dye‐sensitized solar cells (DSCs).^8^ However, the power conversion efficiencies of these devices have not been high enough for commercialization.^9^, ^10^ Recently, perovskite solar cells (PSCs) have attracted wide attention because of their high efficiencies presently achieved in the laboratory >20%,^11^ and there is a strong possibility that an efficiency of 25% will be achieved in the near future.^12^ In addition, there is potential for ultra‐low‐cost production because the major components of device can be deposited with a low‐temperature coating process using inexpensive, abundant materials.^13^

Several kinds of PSC device architectures have been developed.^14^, ^15^, ^16^ One is mesoscopic structures derived from DSCs.^17^, ^18^, ^19^ The other is inverted planar device structures demonstrated by organic photovoltaics researchers.^14^, ^20^ The components of the devices, such as the charge transport layer and electrode, and the processes of fabricating the different device structures are very different.^21^, ^22^, ^23^, ^24^, ^25^, ^26^ To produce a full printing structure, an extremely low‐cost “humble process” has been developed with using a series of cheap materials, but cell efficiencies are still <15%.^27^, ^28^, ^29^ In contrast, the fabrication of champion efficiency (around 20%) PSCs usually require relatively expensive materials, fine control of morphology for each layer, and a vacuum deposition process, defined as a “noble process”.^30^, ^31^ This trade‐off between the performance of the cell and process complexity leads to difficultly in making decisions with respect to the module structure and manufacturing process. A detailed cost estimate is therefore not yet carried out, even though PSCs are expected to be ultra‐low cost devices.

In this work, we first assessed manufacturing costs by analysing two representative PSC modules designed based on the full printable structure with “humble process” to produce moderately efficient modules, and the other based on a precise structure and “noble process” to produce highly efficient modules. We calculated the module cost and carried out a sensitivity analysis of module cost variation relative to efficiency of two kinds of modules. We found that the calculated module costs for PSCs were one third of cost of bulk silicon PV technologies. The levelized cost of electricity (LCOE) was estimated based on module cost and the other parameters reported recently. The LCOE was expected to be competitive with electricity produced from fossil fuels and to be as low as the aggressive target of 6 US cents/KWh proposed in the “U.S. SunShot Initiative” if the module efficiency and lifetime exceeded 12% and 15 years, respectively.

## 2 Designs of PSC Modules and Manufacturing Lines

**Figure 1:**
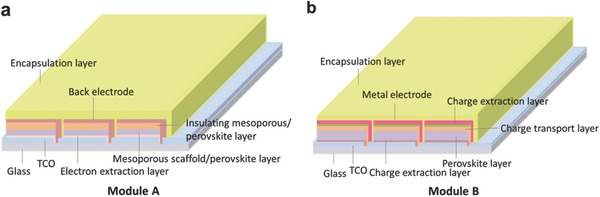
Module schematic diagram of a, Module A with printable mesoporous structure and b, Module B with planar inverted structure. Only part of the module is shown. The relative dimensions of the schematic diagram do not accurately reflect the dimensions of the module. The actual dimensions are described in Table S1.

**Figure** **1** shows the structures of the designed modules, which were assembled with series connections. The cell in module A based on a mesoporous structure can be fabricated by using a series of simple techniques based mainly on screen printing (denoted as humble process) to produce moderately efficient modules as high as 15% (Figure 1a).^29^ Spray pyrolysis can be used to deposit a compact bottom layer such as TiO_2_ on a transparent conductor oxide (TCO) glass on which a pattern of rectangles has been etched with a laser, and the scaffold layers, including the back electrode, can be simply formed by multiple screen printing (with a defined mesh size for producing the desired patterns) and sintering at 400–500 ºC. The perovskite material is dip‐coated within the mesoporous scaffold by moderate thermal annealing (90–150 ºC). Because of the imprecise boundaries produced by the screen printing technique, the active area in module A is unlikely to be very large. Assuming that the active area covered 80% of a module surface with an area of 1 m^2^, we calculated that the module efficiency would be 12% (cell efficiency of 15%) and that a power output of 120 W could be achieved with 1 m^2^ of module A (Table S1). The cells in module B (Figure 1b) based on a precise structure were composed of several layers of high‐quality thin films to produce highly efficient modules ≈20%.^32^, ^33^ Their fabrication required a series of finely controlled processes, production of patterns with lasers, and vacuum evaporation to produce metal electrodes. The high precision of the fabrication processes may cause the manufacturing cost to increase. The narrowness and precision of the etching produced by lasers is expected to improve the accuracy of the boundary and lead to a relatively large active area (0.95 m^2^ in one piece of 1 m^2^ module B). The calculated module efficiency is 19% (cell efficiency of 20%), and the resulting power output is 190 W (Table S1).

## 3 Estimation of Costs of PSC Modules

**Figure 2:**
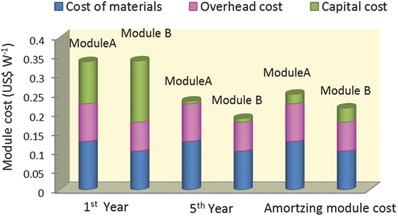
Calculated modules costs of PSC for first year, fifth year and amortize over 5 years with taking depreciation and amortizing capital cost into consideration. The depreciation rate was 50% per year and the capital cost was assumed to remain constant after the five‐year period.

**Figure 3:**
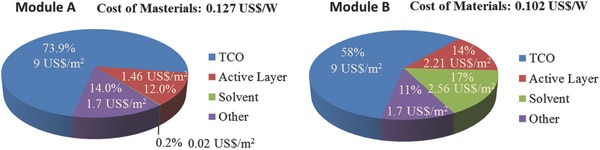
Cost of materials distribution for Module A (left) and Module B (right). The values of materials cost are assumed by the real amount of material used in both strucuture and wholesale price. The 80% materials usage ratio has been considered.

**Figure** **2** shows the costs of modules of Module A and Module B at 1^st^ year, 5^th^ year and amortizing capital cost over 5 years. The module cost can be divided by the cost of materials, overhead cost, and capital cost. The capital costs for Module A and B were calculated based on the capital costs of DSCs fabricated using the printing process and thin‐film silicon solar cells, respectively (Table S2 and S3). The cost of materials was estimated based on the amount of the materials that were used. The overhead cost was estimated based on reasonable assumption. The details of the calculation are shown in the Methods section and Supporting Information. The relatively high module cost in the first year was due to the high depreciation rate (50%) of capital investment. The calculated capital costs in the first year were 0.110 and 0.160 US$/W for Module A and B, respectively. The initial capital cost of Module A was lower because the capital investment associated with use of cheap printing facilities was lower than that of the high‐vacuum machines used in Module B. However, the capital cost rapidly decreased because of depreciation, the result being a monotonic decrease of the total module cost during the first 5 years (Table S4 and S5). After that time, the contribution of capital cost to total cost became very low, so that, the module cost was determined mainly by overhead and materials costs. Regarding the cost of materials, **Figure** **3** presents the distribution of the materials cost for PSCs production routes. Active layers represent of perovskite layer, charge extraction layers, charge transport layer and back electrode. The cost of others in Figure 3 mainly included the expense on sealing materials and glass covered on backside of device. The cost of TCO is tallied separately because it takes up most of the materials cost in both structure. Although inexpensive transport layers and printable electrode materials were used in Module A, the total calculated cost of materials for Module A 0.127 US$/W was a little higher than the cost for Module B 0.102 US$/W (Table S6). The higher cost of materials for Module A was due to the fact that the cost of active‐layer materials (except for TCO) accounted for only a small proportion of the total cost of materials, and the cost of TCO was high due to the smaller active area and low efficiency of Modules A. This result suggests that high efficiency of module can reduce the cost of materials due to enhance the utilization of materials. The overhead costs of Module A and Module B (shown in Table S7 and S8) were estimated to be 0.098 US$/W and 0.075 US$/W based on the report of DSCs and thin‐film silicon solar cells production.^34^, ^35^ Hence, the conclusion could be drawn that cost of Module B produced by Module B and that of Module A produced by Module A are almost the same.

To compare the module cost with other PV technologies and calculate the electricity generating cost, amortizing module cost was also calculated by amortizing total capital cost by working lifetime of equipment. As shown in Figure 2, the amortizing module costs were calculated to be 0.250 US$ for Module A and 0.215 US$ for Module B, which are one third of module cost of bulk silicon solar cells (Table S9). These two amortizing module costs will be used for following sensitivity analysis and estimation of levelized cost of electricity which is usually considered as electricity generating cost.

## 4 Sensitivity Analysis of Module Cost

**Figure 4:**
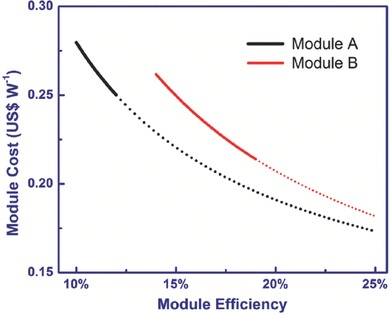
Module cost of PSCs as a function of module efficiency. Except for the independent variables in these Figures, the other parameters associated with Module A and Module B were fixed. The solid lines were calculated based on the range of reported efficiencies; the dashed lines are based on calculations assuming high module efficiencies that are expected but not yet achieved.

It is noteworthy that these cost estimates were based on assumptions about the two kinds of cell structures. However, the assumed parameters may vary when the PSCs are commercialized. Hence, we performed further sensitivity analyses to consider the effect of PCEs on module costs. The module costs increased exponentially as their module efficiency decreased (**Figure** **4**). The part of solid line was corresponding to the efficiency of present research status. The efficiency of Module A was assumed to be 10–12% based on a current cell efficiency of 12–15%. The corresponding estimated module cost was 0.28–0.25 US$/W. The cell efficiency of Module B has been reported to currently be 15–20%, which can result in a module efficiency of 14–19%. The calculated module cost was 0.26–0.21 US$/W. If we further extend the solid line, the module costs of Module A and Module B are getting closer under the same module efficiency (dash line of Figure 4). This result revealed that the module efficiency acted as an important factor for module cost no matter which route was used for manufacturing. Improvement of the cell efficiency and active area by upgrading precision of printing method for further increase of the module efficiency is effective way to reduce the cost of module A.

## 5 Levelized Cost of Electricity Produced with PSCs

**Figure 5:**
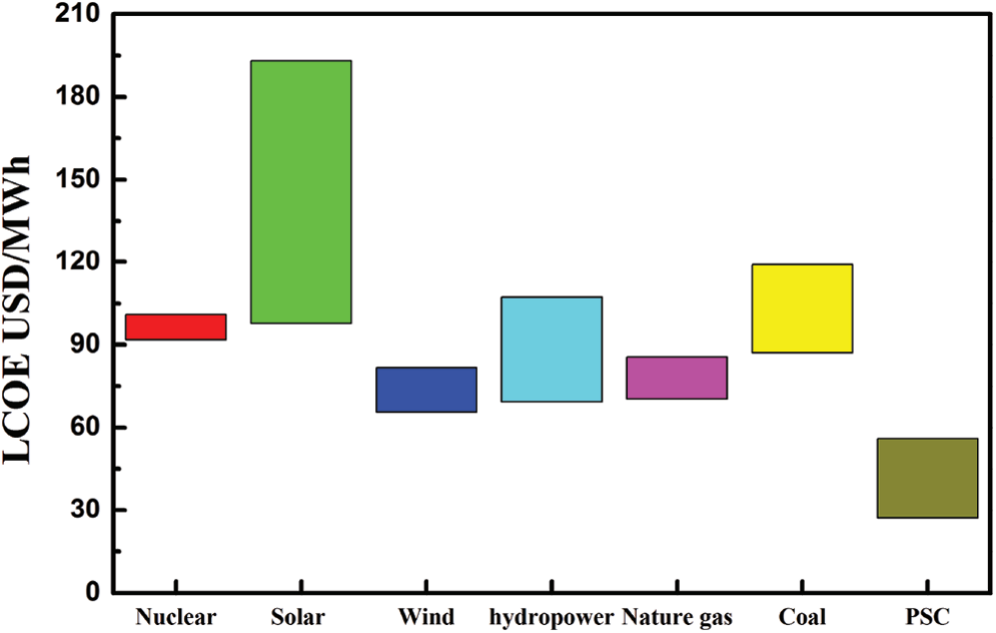
The comparison of LCOE based on coal, nature gas, unclear, wind, commercialized solar PV, hydropower and PSC. The LCOE values are referenced to the Levelized Cost and Levelized Avoided Cost of New Generation Resources in the Annual Energy Outlook 2015 reported by United States Energy Information Administration.

The LCOE is typically used to compare system costs of electricity produced using different sources of energy. The LCOEs of traditional energy sources were 7.04–11.90 US cents/kWh, and the costs of solar PV technologies were 9.78–19.33 US cents/kWh reported in Levelized Cost and Levelized Avoided Cost of New Generation Resources in the Annual Energy Outlook 2015.^36^ The LCOE was calculated according to Equation. (2) (Method part), it was affected mainly by module cost, efficiency, and lifetime. In our module cost analysis, both Module A and Module B were estimated to produce perovskite solar modules at a cost in the range of 0.21–0.28 US$/W. We calculated the LCOE of a perovskite solar module by assuming a module cost of 0.25 US$/W and a lifetime of 15 years. The LCOEs were 4.9 US cents/kWh, 4.2 US cents/kWh, and 3.5 US cents/kWh corresponding to module efficiencies of 12%, 15%, and 20%, respectively, which were lower than that of traditional energy sources (**Figure** **5**). Details of the calculation are shown in the Methods section and Table S10. This analysis indicates that module efficiency has a significant influence on the LCOE.

**Figure 6:**
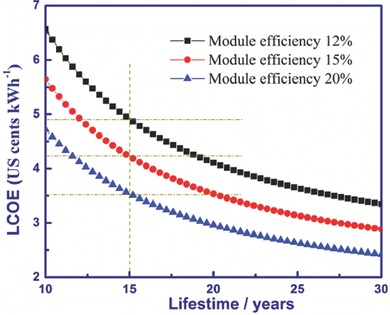
The relationship between LCOE and lifetime. A system lifetime <10 years was not considered in our analysis.

**Figure** **6** shows the effect of lifetime on the LCOE of perovskite solar cells. The LCOEs estimated by module efficiency of 12%, 15% and 20% decrease exponentially with the extension of the system lifetime in the range 10–30 years. For high‐efficiency (20%) modules, a lifetime of 10 years can lead to an LCOE of 4.7 US cents/kWh. The low‐efficiency (12%) modules require a long lifetime (30‐years) to achieve the similar LCOE. A conservative estimate of discount rate 5% is used above.

Based on the analysis above, the module efficiency and lifetime were the most sensitive factors for the LCOE of PSCs. The ultra‐low LCOE of PSCs was achieved to be 3.5–4.9 US cents/kWh with 15 years lifetime, surpassing the United States “SunShot Initiative” target of 6.0 US cents/kWh.^37^ Although the efficiency of small size PSCs had already beyond 20%, the efficiency record of larger size with 1 cm^2^ aperture area was reported to be 15%. Regarding lifetime, studies related to long‐term stability of PSCs were still limited.^38^, ^39^ Hence, improvement of the efficiency and the lifetime of PSCs are urgent tasks from the perspective view of cost, and more efforts should be devoted to this field.

## 6 Conclusions

We designed two representative module structures for PSCs as low materials cost module and a high precision high efficiency module. The costs of the modules were estimated based on an annual capacity of 100 MW and the reported solar cell performances of the two kinds of cell structures. We found that the module costs for both structures could be much lower than those of other solar PV technologies. The results of the sensitivity analysis indicated that an increase of module efficiency could significantly reduce module cost. The fabrication of high‐efficiency modules through the high precision processes was the most promising approach for further reducing the cost. The LCOE of perovskite solar cells was also very sensitive to module efficiency and can be expected to be lower than that of traditional energies if the module efficiency and lifetimes can exceed 12% and 15 years, respectively. To achieve these targets, more efforts should be made to improve the lifetime and efficiency of perovskite photovoltaic devices rather than to identify cheaper materials and processes.

## 7 Methods

*Module cost estimation*: To assess the cost of fabricating the modules, we assumed the production capacity of both routes to be 100 MW per year. The module cost was composed of the capital cost, cost of materials, and overhead cost. The capital cost was based on depreciation of capital investment (CI). Because the full process of printing module A was based on the fabrication of dye‐sensitized solar‐cell (DSCs), the capital cost was based on the capacity of DSCs, for which the module efficiency was 6% and the CI was 11 million US$ for a production capacity of 50 MW. Because the module efficiency of 12% via Module A was twice that of DSCs, the capital investment for Module A (CI_Module A_) with 100 MW capacity per year was estimated to be 11 million US$ per year (Table S2). The module cost for Module B was estimated in a similar manner based on the production of silicon solar cells with an annual capacity of 60 MW, as shown in Table S3. The capacity for Module B was 1.6 times the capacity of thin‐film, silicon solar cells because of the higher efficiency of the PSC module. In addition, the capital investment for a capacity of 100 MW via Module B (CI_Module B_) without chemical vapor deposition was 40% of the CI of a thin‐film, silicon solar cell manufacturing line with a capacity of 60 MW. Thus, the CI_Module B_ for a capacity of 100 MW was 16 million US$. The details of the estimate are presented in the Supporting Information.

The depreciation of the facility resulted in a decrease of capital investment from year to year according to Equation (1):^37^

(1) $$\mathrm{CI}(n)\;=\;\mathrm{CI}\;\times \;\beta^{n}$$

where *n* is the number of years after construction and β is the depreciation ratio, which we assumed to be 0.5 based on the PV industry. Depreciation of an investment should cease when *β^n^* is less than 0.1. After four years, there was no further depreciation of the investment because (0.5)^4^ = 0.063. The capital costs of Module A and B were based on the ratio of capital investment to power output, which changed from 0.110 to 0.007 US$/W and from 0.160 US$/W to 0.010 US$/W, respectively, during the first five years (Table S4, Table S5). The module cost that was used for comparisons with other PV devices was calculated by summing the capital amortization cost, the cost of materials, and the overhead cost. The capital amortization costs for Module A and B were 0.025 US$/W and 0.037 US$/W, respectively, based on the annual worth of CI (2.54 million USD for Module A and 3.70 million USD for Module B); they were equated to [i * (1 + i)^^^n* CI]/[(1 + i)^^^n‐1] where i is annual interest and n is 5‐year equipment lifetime. The estimate of annual interest 5% is used above as assumption value in 2020. It is quite possible because the global economy is experiencing low interest rate age.

The costs of materials for Module A and B were estimated to be 0.127 US$/W and 0.102 US$/W, respectively, based on the ratio of investment in materials to power output with usage percentages of materials of 80%. The calculation prices were referring to the other devices.

The overhead costs consisted of labor costs, the cost of renting facilities, and the cost of utilities. The labor cost of 0.0304 US$/W estimated was based on the PV industry average (Table S7). Based on DSCs and thin‐film, silicon solar cell manufacturing lines, the rents for Module A and B were estimated to be 0.035 US$/W and 0.022 US$/W, respectively, and the costs of utilities for Module A and B were estimated to be 0.035 US$/W and 0.022 US$/W, respectively. After adding 1% of the capital costs for maintenance fees, which were 0.085 million US$/year and 0.012 million US$/year for Module A and B (**Table S9**), the overhead costs of Module A and B were calculated to be 0.098 US$/W and 0.075 US$/W, respectively (Table S8).

The resultant module costs calculated based on our assumptions were 0.250 US$/W and 0.214 US$/W for Module A and B, respectively (Table S9). These were the baseline values used in the sensitivity analysis.

*Estimation of the levelized cost of electricity*: The total cost of the solar cell system, including the costs of the module, balance of systems (BOS), land, support structures, wiring, power conditioning, and installation,^37^ was calculated with Equation (2):^40^, ^41^

(2) LCOE=(ICC × 1000 CRF)/(CF×8760)+O & M

where ICC is the Installed Capacity Cost ($/W DC) = BOS cost + module cost,

CRF is the Capital Recovery Factor = (*i* × (*i* + 1)*^n^*)/((*i* + 1)*^n^* ^– 1^),

CF = Alternating Current Capacity Factor (0.8 × sunlight/8760 hours, reduced by 20% losses to go from direct current to alternating current),

O&M = Operation and Maintenance ($/kWh),

*i* = discount rate,

*n* = lifetime (the lifetime of system).

Assumptions were as follows: BOS was 75 US$/m^2^ based on based on the projected long term goal for traditional silicon‐based solar cell in 2020.^37^ BOS costs for efficiency of 12%, 15% and 20% were 0.625 US$/W, 0.5 US$/W and 0.375 US$/W, respectively, by using BOS cost = 75 US$ × m^–2^/output; O&M = $0.001/kWh; *i* = 5%, and *n* = 20 (no tax credits and no accelerated depreciation), from these values, CRF (*i* = 5%, n = 15) = 0.096. To find the energy produced in a year by 1 W of installed PV, we used a CF of 20%. This assumption takes into account that PV cells only operate at a fraction of peak power when averaged over the course of a year with 1700 kWh/m^2^ per year.^42^

## Supporting information

As a service to our authors and readers, this journal provides supporting information supplied by the authors. Such materials are peer reviewed and may be re‐organized for online delivery, but are not copy‐edited or typeset. Technical support issues arising from supporting information (other than missing files) should be addressed to the authors.

Supplementary
