# Supplementary material for: Cost‐Performance Analysis of Perovskite Solar Modules
Source: Adv Sci (Weinh). 2016 Sep 15;4(1):1600269. doi: 10.1002/advs.201600269 (PMC5238749; doi:10.1002/advs.201600269)
Supplement: Supplementary file 1 — Supplementary [file ADVS-4-0-s001.pdf]

Copyright WILEY-VCH Verlag GmbH & Co. KGaA, 69469 Weinheim, Germany, 2016.

ADVANCED SCIENCE
Open Access

Supporting Information

for *Adv. Sci.,* DOI: 10.1002/advs.201600269

Cost-Performance Analysis of Perovskite Solar Modules

*Molang Cai, Yongzhen Wu, Han Chen, Xudong Yang, Yinghuai Qiang, and Liyuan Han**

Supporting Information

Cost-performance analysis of perovskite solar modules

*Molang Cai,[a] Yongzhen Wu,[a] Han Chen,[b] Xudong Yang[b], Yinghuai Qiang[c] and Liyuan Han[a,b]**

Module designing and module manufacturing processes for Module A and Module B

Table S1. Designed parameters of Module A and Module B.

module parameters	Module A	Module B
module size	$1m^2$	$1m^2$
distance between cells	0.2cm	0.2mm
margin areas at left and right sides of module	0.9 cm	1.05cm
margin areas at up and down sides of module	0.5 cm	0.5 cm
size of rectangular cells	1m×1cm	1m×1cm
number of cells on one piece of module	82	96
active area on one piece of module	$0.81m^2$	$0.95m^2$
cell efficiency	15%*	20%*
module efficiency	12%	19%
power output of one piece of module	120W	190W

* We assume that the cell efficiency of module A and module B are 15% and 20% respectively according to the reported highest efficiency based on full printing and high performance PSCs.

Estimation of Module cost and Levelized cost of electricity (LCOE)

Table S2 Comparing processes of dye sensitized solar cells (DSCs) with PSCs and estimation of capital cost for Module A.

	Processes
Common used in both manufacturing lines of DSSC and Module A	Substrate clean and etching, Screen printing and dip-coating, Sintering and drying, Encapsulation and sealing, Electrical interconnection
Only used in manufacturing line of DSSC	Hole drilling and sealing, Electrolyte filling and Framing, Sandwiching of electrodes
Only used in manufacturing line of Module A	Spraying operation
Capital investment of 50MW DSSCs production line	11 million US$
Estimated capital investment of 100MW PSCs production line	11million US$

To assess the manufacturing line, assuming that production capacity (*C*) of Module A is 100 MW. Eq. 3 presents the parameters of production capacity:

$C = P * V * T$ (Eq. S1)

where here P is the power output of one piece of PSCs module, based on output per square meter of 1000 W, P is defined as P = 1000* η_{module} (Eq. 4), the calculated value of P (Module A) is 120W; T is the producing time by 350 days/year (504000 min/year), V is the producing rate calculated as 100MW/(504000min*120W/m^2)=1.654 m^2/min.
As an important factor of capacity, producing rate highly depends on the technologies used for the manufacturing line.
Since the full printing mesoporous structure is derived from DSCs, the capacity of Module A is compared with that of DSCs manufacturing line to assume capital investment of Module A. Table S2 shows that the technologies and facilities of DSCs[24] and Module A manufacturing line are very similar. In both cases, sintering the film to high temperature takes longer time compared to other processes as rate dominating step. This slowest sintering rate is considered as producing rate equal to the value of 1.654 m^2/min calculated above. The module efficiency of DSCs is around 6%[25] resulting P (DSCs) of 60W/m^2 and P(Module A) is 120W/m^2. According to Eq.S1, capacity of Module A (C(Module A)) is two times of capacity of DSCs manufacturing line (C(DSCs)) with same capital investment. Thus, estimated capital investment of 100MW Module A is 11 million US$, which is same as that of 50MW DSSCs production line.

Table S3 Comparing processes of silicon solar cell with PSCs and estimation of capital cost for PSCs Module B.

	Processes
Common used in both manufacturing lines of thin film silicon solar cells and Module B	Substrate clean and etching, Laser ablating for active layers, vacuum deposition for metal electrode
Only used in manufacturing line of Si thin film solar cells	Active layer deposition using PECVD
Only used in manufacturing line of Module B	Doctor blend active layers
Capital investment of 60 MW Si thin film solar cell production line	26 million US$
Estimated Capital investment of 100 MW PSCs production line	16 million US$

We suspect that production capacity of Module B (C (Module B)) is 100 MW to assess the manufacturing line. According to Eq. 3, producing rate of Module B (V (Module B)) is calculated as

100MW/(504000min*190W/m^2) =1.044 m^2/min.

Since the planar inverted structure is derived from the thin film silicon solar cells, capacity of Module B is estimated refer to the commercial data of thin film silicon solar cell online. Table S3 compares the common processes and differences between thin film silicon solar cell and Module B. Evaporation deposition of back electrode is considered as rate dominating step in manufacturing line. The module efficiency of thin film silicon solar cells is 12% with power output of 120W/m^2 for silicon solar cell.[20] The power output of Module B is 190W, which is 1.6 times higher than thin film silicon solar cell. According to Eq. 3, the capacity of Module B is around 1.6 times to capacity of thin-film silicon solar cell manufacturing line. In addition, the costly facility PECVD is taking nearly 40% of capital investment is only used in Si thin film solar cell. Thus, the capital investment of 100MW PSCs produced by Module B is 16 million US$, which is sum of 60% of capital investment of 60MW thin film silicon solar cell system and other equipments only used in Module B.

Table S4. Capital cost of Module A along with facility depreciation

Year	investment million US$	rate of depreciation	depreciation percentage	Capacity MW	capital cost US$/W
1st	11.00	50%	100%	100	0.110
2nd	5.50	50%	50.0%	100	0.055
3rd	2.75	50%	25.0%	100	0.028
4th	1.38	50%	12.5%	100	0.014
5th	0.69	50%	6.3%	100	0.007
after 5th	0.69	-	-	100	0.007

Table S5. Capital cost of Module B along with facility depreciation

Year	investment million US$	rate of depreciation	depreciation percentage	Capacity MW	capital cost US$/W
1st	16.00	50%	100%	100	0.160
2nd	8.00	50%	50.0%	100	0.080
3rd	4.00	50%	25.0%	100	0.040
4th	2.00	50%	12.5%	100	0.020
5th	1.00	50%	6.3%	100	0.010
after 5th	1.00	-	-	100	0.010

Capital depreciation is an important tool for businesses to recover certain capital costs over the property's lifetime. Allowing businesses to deduct the depreciable basis over five years reduces tax liability and accelerates the rate of return on a solar investment. This has been a significant driver for the solar industry and other energy industries.

Table S6 Estimation of materials cost for Module A and Module B.

	Module A	Module B
expected materials cost US$/m^2	12.18	15.47
materials use ratio	80%	80%
actual materials cost USD/m^2	15.23	19.34
module output W/m^2	120	190
materials USD/W	0.127	0.102

A simple, but important arithmetic relationship underlies the analysis. Most PV costs are given in dollars per watt peak (US$/W). This is fine for the end user (especially if it is a system price), but it hides the nature of the technical challenges, especially in thin films. Two components go into a cost in US$/Wp: the output or efficiency of the device; and its manufacturing cost per unit area. By combining them you get a cost in US$/Wp. The actual relationship is very simple: the dollars per watt cost can be found simply by dividing the manufacturing costs per unit area (say US$/m^2) by the output of the same area (which for a m^2 is 1000 Wp/m^2 times the efficiency). The same relationship works at the module level: the module cost (in US$/module) divided by its output (Wp/module) is its US$/Wp cost. Obviously, the same relationships show how to go the other way: if one knows the US$/Wp cost and either the efficiency (or unit output) or the area cost, one can calculate the missing parameter. The simple relationship is as follows Eq S2:

US$/Wp = (Cost/unit area) / (output/unit area). Eq. S2

Unit area can be the module area; or the cost per square meter. Output per square meter is 1000 Wp/m^2 times the efficiency. The materials cost is calculated as following: expected materials cost divided by module output and materials use ratio, for Module A is 12.18/(120×0.8), for Module B is 15.47/(190×0.8).

Table S7. Labor cost of manufacturing line for PSCs Module A.

	number	Average wage US$/year	cost US$/year
employee	76	40000	3040000
	labor cost US$/W		0.0304

One manufacturing line requires three groups switch every 8h for one day, and one group is ready for switch on holidays. There are 15 operators in one group; 12 technicians and 4 managers to direct the operators, the number of employee is 76 in total. The labor cost is 0.0304 USD/W for year estimated in table S5. The average wage is assumed by considering the balance between developing country such as China and developed country such as USA.

Table S8 Estimated overhead cost for Module A and Module B.

Cost component	PSC Module A Cost (US$/W)	PSC Module B Cost (US$/W)
Facilities (e.g. rent)	0.033	0.021
Utilities (electricity, water)	0.033	0.022
Labor	0.0304	0.0304
Maintenance	0.0011	0.0016
Sum	0.098	0.075

The overhead cost is estimated by sum of facilities, utilities, labor and maintenance fee.

Table S9 Estimation of module cost for PSCs.

Year	capital cost US$/W	Cost of materials US$/W	overhead cost US$/W	module cost US$/W
Module A	0.025	0.127	0.098	0.250
Module B	0.037	0.102	0.075	0.214

The module cost is the sum of capital cost, materials cost and overhead cost for both Module A and Module B.

Table S10 Calculation of levelized cost of PSCs based on different module efficiency.

Module efficiency	12%	15%	20%
Module cost US$/W	0.25	0.25	0.25
LCC	0.88	0.75	0.63
CRF	0.096	0.096	0.096
CF	0.20	0.20	0.20
O M	0.001	0.001	0.001
LCOE US cents/kWh	4.9	4.2	3.5

[1] G. Smestad, C. Bignozzi, R. Argazzi, Solar Energy Materials and Solar Cells **1994**, 33, 253.
[2] T.-C. Wei, S.-P. Feng, Y.-H. Chang, S.-J. Cherng, Y.-J. Lin, C.-M. Chen, H.-H. Chen, Int. J. Electrochem. Sci **2012**, 7, 11904.
